# Moral development in personal and professional identity formation: Longitudinal impact of teaching the role of medicine during the Holocaust in health professions education

**DOI:** 10.3205/zma001830

**Published:** 2026-03-23

**Authors:** Hedy S. Wald, Madelin S. Riesen, Claudia Kiessling

**Affiliations:** 1 Warren Alpert Medical School of Brown University, Department of Family Medicine, Providence, Rhode Island, USA; 2 Witten/Herdecke University, Faculty of Health, Witten, Germany; 3 Witten/Herdecke University, Faculty of Health, Chair for the Education of Personal and Interpersonal Competencies in Health Care, Witten, Germany

**Keywords:** Professional Identity Formation, moral development, medical education, medicine during National Socialism, medicine during the Holocaust

## Abstract

**Objectives:**

Critically reflecting on history of medicine during Nazism and the Holocaust (MNH) which included egregious ethical violations such as genocide complicity is a powerful platform for scaffolding Professional Identity Formation (PIF) with contemporary relevance for health professions education (HPE), clinical and research practices, and public policy. The study explored the longitudinal impact of MNH curriculum with an Auschwitz Memorial study trip on personal and PIF including moral development as a key PIF component.

**Methods:**

The authors used immersion-crystallization qualitative thematic analysis to analyze medical and psychology students’ reflective writings at 6 weeks (N=10) and 1.5 to 2 years (N=24) post 2019 MNH curriculum with study trip.

**Results:**

Four distinct themes were identified, ie. curriculum and study trip impact on me as a 1) person, 2) student & future health professional, 3) member of a health profession (and health system), and 4) global citizen & part of humanity (philosophical/existential aspects). Each theme included five subthemes, ie. moral sensitivity, moral judgment, moral motivation, moral character, and moral outcome. Themes/subthemes were mapped to a conceptual framework for a moral development model within personal and PIF.

**Conclusions:**

This curriculum catalyzed critically reflective learning/meaning-making supporting history-informed personal and PIF, and moral development within HPE. Students described compelling and relevant impact of the MNH curriculum on their perspectives and behavior within their studies, the health care system, and society. MNH curriculum is proposed as a fundamental HPE component cultivating attitudes, values, and behaviors for empathic, morally courageous leadership within inevitable healthcare and societal challenges.

## Zusammenfassung

**Zielsetzung:**

Die kritische Auseinandersetzung mit der Geschichte der Medizin während des Nationalsozialismus und des Holocaust (GMNH), die schwerwiegende ethische Verstöße wie die Mittäterschaft am Völkermord beinhaltete, ist eine wirkungsvolle Plattform für die Förderung der beruflichen Identitätsbildung (engl.: Professional Identity Formation, PIF) mit aktueller Relevanz für das Studium der Gesundheitsberufe (engl.: Health Professions Education, HPE), die klinische und wissenschaftliche Praxis sowie Public Policy. Die Studie untersuchte die langfristigen Effekte des GMNH-Curriculums mit einer Exkursion zur Gedenkstätte Auschwitz hinsichtlich der persönlichen und beruflichen Identitätsbildung, einschließlich moralischer Entwicklung als Schlüsselkomponente der PIF.

**Methoden:**

Die Autorinnen nutzten die qualitative thematische Analyse (Immersions-Kristallisations-Ansatz), um die Reflektierenden Schreiben (engl.: Reflective Writings, RWs) von Medizin- und Psychologiestudierenden sechs Wochen (N=10) und 1,5 bis 2 Jahre (N=24) nach dem GMNH-Curriculum mit Exkursion im Jahr 2019 auszuwerten.

**Ergebnisse:**

Es wurden vier Themen identifiziert: die Auswirkungen des Curriculums und der Exkursion auf mich als 1) Person, 2) Student*in und zukünftige*r Ärzt*in bzw. Psycholog*in, 3) Angehörige*r einer medizinischen Profession (und des Gesundheitssystems) und 4) Bürger*in einer globalen Welt und Teil der Menschheit (philosophische/existenzielle Aspekte). Jedes Thema umfasste fünf Unterthemen: moralische Sensibilität, moralisches Urteilsvermögen, moralische Motivation, moralischer Charakter und moralisches Outcome. Die Themen und Unterthemen wurden in einem konzeptionellen Modell der moralischen Entwicklung innerhalb der persönlichen und PIF dargestellt.

**Schlussfolgerungen:**

Das GMNH-Curriculum förderte kritisch-reflexives Lernen (engl.: Meaning Making), das eine historisch informierte persönliche und PIF sowie die moralische Entwicklung innerhalb der HPE unterstützte. Die Studierenden beschrieben eindrucksvolle und relevante Auswirkungen des GMNH-Curriculums auf ihre Perspektiven und ihr Verhalten innerhalb ihres Studiums, des Gesundheitssystems und der Gesellschaft. Die Autorinnen empfehlen ein GMNH-Curriculum als wesentlichen Bestandteil der Ausbildung der Gesundheitsberufe, das Einstellungen, Werte und Verhaltensweisen für eine empathische, moralisch mutige Haltung innerhalb der unvermeidlichen Herausforderungen im Gesundheitswesen und in der Gesellschaft fördert.

## 1. Introduction

*“A truer and more meaningful educational experience in Holocaust teaching depends on far more than cognitive knowledge: it depends on the internalization of the human and moral dilemmas posed by the Holocaust to modem society and to each of us in the form of conflicting choices and their practical resolution.” *([1], p.353) 

The active, constructive, transformative process of Professional Identity Formation (PIF) with qualities of moral resilience and ongoing critical reflection is a fundamental goal within health professions education (HPE) [2]. Educators are challenged to design formative educational experiences for PIF to support trainees acting as “intentional agents” ([3], p.305). PIF, socially constructed and an integration of personal identity and professional self [3], [4], is “a representation of self, achieved in stages over time during which the characteristics, values, and norms of the medical profession are internalized, resulting in an individual thinking, acting, and feeling like a physician” ([5], p.1447) and establishing core values, moral principles, and self-awareness [6]. PIF is intertwined with moral development [7] on the path to moral competence [8], which is an essential component of PIF [9] in enabling students to cope with inevitable moral dilemmas (and complexities) in medical practice [10].

Our prior research analyzing medical and psychology students’ reflective writings (RWs) revealed that their personal and PIF is supported by studying the history of medicine during Nazism and the Holocaust (MNH) with a curriculum including an Auschwitz Memorial study trip and has contemporary relevance for HPE and practice [11]. This aligns with the value of history-informed PIF, a concept introduced by the Lancet Commission on MNH [12]. Studying the history of MNH is vital given that the PIF socialization process includes not only internalization of characteristics, values, and norms of the medical profession but critical engagement with and examination of them in order to uphold required professional standards to meet obligations to patients and society [12], [13]. Our prior research on the impact of studying MNH with a study trip revealed a sensitized moral compass [14], heightened awareness of contemporary relevance for preventing abuse of power and respecting diversity, and moral intention to apply their learning responsibly within the profession and society [11].

Through this work, we became more interested in moral development as key within PIF on the path to “moral maturity” [7] and “moral competence” with self-representation as a moral agent [8] including moral awareness, moral judgment, and moral practice [15]. Cultivating moral decision-making, including moral reasoning and other related processes [16], [17] and embodying moral principles to guide one’s actions independent of pressure from social norms [18] is essential for preserving human dignity within patient care, research, and public policy [15].

Rest [19], [20] proposed moral behavior as the product of four independent psychological subprocesses, not necessarily in a linear sequence as discussed by Bebeau and colleagues [21]:

1. moral sensitivity, ie. recognition of a moral situation and how our actions affect other people, with self-awareness of one’s own role and responsibility [22];
2. moral judgment, ie. evaluating an action as morally justified or not;
3. moral motivation, ie. prioritizing moral values over other personal values, and
4. moral character, ie. “sufficient perseverance, ego strength, and implementation skills to be able to follow through on his/her intention to behave morally” ([23], p.203).

Despite the first three processes, according to Bebeau and colleagues, moral failure will occur if lacking in moral character [24].

Ideally, the process of PIF can support internalizing principles and values such that actions flow habitually from one’s moral compass [25]. In Kegan’s view [4], the highest stage of identity formation is becoming a person who chooses, internalizes, and lives by good moral values.

Stagnation in moral development, regression, and even moral decline or “erosion” however, occurs during HPE [7], [26], [27], [28], [29], [30], [31], [32] and best practices for cultivating moral competence are thus needed. A medical ethics teaching tools intervention did not lead to increased moral competence [33] and it is unclear why some clinical learning activities may enhance or rather hinder moral development [34]. Focus in ethics courses on codes and regulations with less or absence of promotion of moral reasoning and moral decision-making skills has been noted [35] and some have questioned whether teaching ethical virtues is plausible at all [31].

Moral competence is an area that young practitioners feel unprepared for and can be associated with moral distress [15], [36]. Higher levels of psychological distress and burnout are associated with a weaker sense of professional identity [37]. In line with our work, medical or health humanities curriculum promotes wellness and resilience [38] and has value for promoting critical thinking including openness to new perspectives and empathy with intentional reflective exercises [38], [39], [40]. Teaching MNH history in the humanities including a study trip to Auschwitz fosters bioethical reflection in medical students [41] and supports moral responsibility [42] and PIF [43], [44] in medical and nursing trainees. Medical ethics education can purportedly be more effective utilizing the power of this historical narrative including place-based learning [45]. While not a direct comparison due to different populations, impact studies of study trips to Holocaust historical sites for secondary and high school students have revealed changes in attitude, e.g. increased level of perspective taking and identification with Jews [46] and enhanced awareness of the Holocaust, racism, human rights and citizenship values [47]. Effectiveness studies from the field of memorial pedagogy (not including health professions students) showed mixed results regarding long-term effects of site visits as well as limited impact on xenophobic or other extremist attitudes, understanding historical contexts, self-reflection, and personal relevance to one’s own life and the present [48], [49]. There may well be different effects on adults’ learning using site visits with working professionals and people in training [50]. According to Liu and colleagues in regard to HPE, “a deeper understanding of moral development is long overdue” ([18], p.1004). 

Although international and German medical faculties in cooperation with memorial sites offer programs specific for health profession students [41], [51], [52], [53], longitudinal impact of learning and reflecting on this history on personal and PIF of health profession students including moral development within HPE has, to our knowledge, not been studied. We thus undertook a study of the longitudinal impact of a MNH curriculum, including an Auschwitz Memorial study trip component, on medical and psychology students.

## 2. Methods

### 2.1. Setting and study population

The study was conducted as a follow-up study after a 3-year interprofessional curriculum at Witten/Herdecke University (UW/H) entitled “Cultivating Medical Awareness and Ethics through the Example of Medicine in National Socialism” [11], [54]. Students were able to participate in any or all years of the course which is a special study module (“Studium Fundamentale”) for all UW/H students. We have previously described the course objectives [11]. In addition to history seminars, the curriculum included individual and small group reflection on survivor documentaries, assigned readings [55], [56], [57] and study trips to memorial sites (e.g. Auschwitz Memorial, Buchenwald Memorial, Hadamar Memorial). The 2019 study program included a study trip to the Auschwitz Memorial with additional implementation of interactive RW to support meaning-making and transformative learning [58]. 

Forty-six students (39 medical, 7 psychology) in various study years participated in the 2019 course and four-day study trip (100% of course participants on the trip). Students were invited to four voluntary RW sessions each evening. We have described the value of using interactive RW in the curriculum and findings of the qualitative analysis of these RWs i.e. RW1 to RW4 [11].

### 2.2. Data collection and data management

Six weeks after the study trip (June 2019), students participated in a brief RW session to reflect on their trip experiences and the course (RW5). They were invited to voluntarily submit these RWs to the course leaders for further content analysis. Eleven students submitted their RW5 (24% of the cohort). RW of author MSR as a curriculum participant was excluded.

In October 2020, students were contacted by author MSR (medical student and curriculum participant) to participate in the follow-up study and invited to again reflect on their course and trip experiences within RW (RW6). Students submitted their RWs to MSR either by email or in person. 24 students (52% of the cohort) submitted their RW6. 

Self-selected identifier codes were converted into consecutive ID numbers (e.g. 1.5). Various demographics were not collected to maintain anonymity. All RWs were translated into English by a professional translator to enable all researchers’ participation in the analyses. 

### 2.3. Data analysis 

The research team included a sixth-year medical student (MSR), a professor of family medicine/clinical psychology with experience in interactive RW, PIF, and qualitative research (HSW), and a medical education professor with research experience in qualitative analysis and curriculum development and a dissertation on medicine during National Socialism (CK). We conducted qualitative thematic analyses [59] of the students' RWs, using the English translation by all researchers. The interprofessional, intergenerational research team contributed expertise to the data analysis, including relevant researcher characteristics and reflexivity to the qualitative assessment. [60], [61]. Our team reflected on how cultural and family backgrounds might influence data analysis, including HSW, who acted as an external auditor for the data analysis, e.g., two German researchers and one U.S. researcher. We discussed cultural and linguistic interpretations of the RWs, consulting the translator when necessary. We used an inductive approach to analyze and interpret data. Two authors (MSR, CK) independently analyzed all data using immersion/crystallization method, reading and rereading narratives with “cognitive and emotional engagement”, resulting in “intuitive crystallizations” to identify and extract subthemes to saturation [60], [62]. Reflexivity was a core part of our data analysis, including awareness of the researcher’s subjectivity. We discussed how biases might influence data interpretation. MR was one of the students attending the excursion and guided the reflection process by her experience during the excursion [61]. Reaching consensus while giving voice to the student researcher was essential.

We analyzed ten RWs (RW5) for theme piloting, with discrepancies resolved through discussion. The remaining RWs (RW6) were analyzed in a sequence of two batches of twelve RWs and then discussed. In analyzing the RWs of RW6, we (MSR, CK) noticed that these RWs were longer, richer in terms of reflective processes and addressed slightly different themes than the RWs of RW5. These observations that resulted from the data analysis and interpretation of MSR and CK were shared and discussed with HSW, who acted as an external auditor for the data analysis. The research team decided to focus the thematic analysis primarily on the RWs of RW6 and then returned to the RWs of RW5 to compare and contrast extracted themes. Data analysis then continued until thematic saturation was reached, as determined by research team consensus. MSR and CK worked with the final subthemes in iterative cycles to reach consensus on grouping the subthemes into thematic themes to be mapped onto a conceptual framework of moral development. Again, HSW acted as external auditor for the development of the thematic framework, reviewing the entire thematic analysis and the moral development framework to ensure trustworthiness and credibility. Any disagreements were resolved by several video conference discussions to reach consensus on final subthemes.

## 3. Results

### 3.1. Conceptual framework

**Figure 1:**
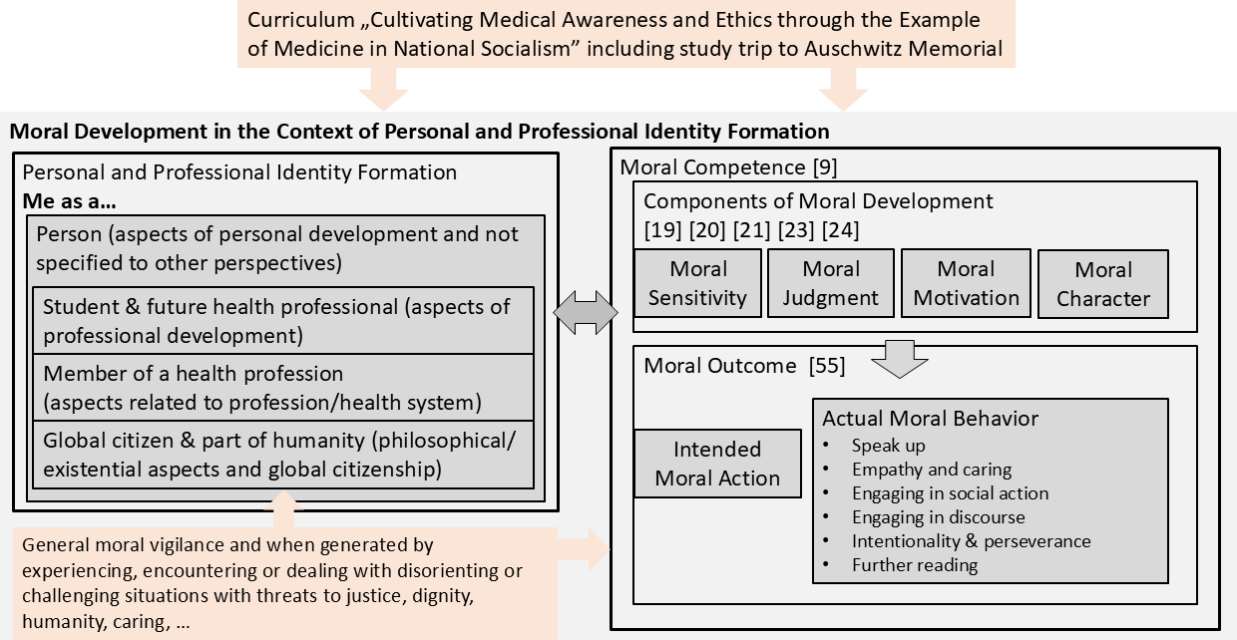
Conceptual framework

Students’ RWs of the follow-up study focused predominantly on moral development topics in the context of personal and PIF. The iterative process of analyzing and discussing themes and subthemes therefore included discussions about existing models of moral development and moral competence and how these models resonate with personal and PIF, guiding re-evaluation of extracted themes and subthemes as well as the overall framework (see figure 1 (Fig. 1)). These themes were found to be consistent with the work of Rest [19], [20], Bebeau and colleagues [23], Bazzetta [63], and Lind [8], [64]. 

As the students described the curriculum and the study trip experience impacting their personal and PIF, they expressed various perspectives on how they interact with the world and reflected on themselves as a person, as a student and future health professional, as a member of a health profession, and as a global citizen and part of humanity. Aspects of moral sensitivity, moral judgment, moral motivation, and moral character were expressed for each of these perspectives [19], [20], [23]. They also reported intended moral actions and actual moral behavior, defined as moral outcomes by Bazzetta [63] and Lind [8], [64]. In general, these moral actions were concretely and specifically described, often emerging in the context of experiencing or dealing with disorienting or challenging situations that threatened justice, dignity, humanity, or caring. Examples are provided in table 1 (Tab. 1).

### 3.2. Themes and subthemes

Perspectives on the curriculum and study trip experience in the context of personal and PIF were identified as themes. Self-reflection (theme 1) related to personal aspects not specific to their studies, profession or society. Theme 2 was reflection on their identity as a student and future health professional. We also extracted reflections about the profession in general and the health care system, and the students’ role within these (theme 3), as well as about their role in society, with existential questions about justice, dignity or humanity (theme 4). These reflective statements of themes 3 and 4 were often formulated as “we as ...” or third person statements (“the profession ...”) rather than using “I” or "me" as in themes 1 and 2. For each of the four themes, the above-mentioned morality components [19], [20], [23] and moral outcomes [63] were defined as subthemes (subthemes a-e). The RWs did not always include all subthemes. Some differences in topic focus between RW5 and RW6 are elaborated in section 3.3. Subtheme titles are presented in italic. Illustrative quotations are presented with quotation marks. Additional illustrative quotes are summarized in table 2 (Tab. 2), table 3 (Tab. 3), table 4 (Tab. 4) to table 5 (Tab. 5).

#### 3.2.1. Theme 1: Curriculum and study trip impact on me as a person 

Students described an enhanced *moral sensitivity* for what happened during National Socialism: “I have become much more sensitive/aware of the whole issue.” (2.5) and an enhanced awareness of any forms of injustice or discrimination today: “Since then I am clearly sensitized for subtle processes of such objectifications, devaluations, coercive measures or even human attention in desperate situations.” (52.6). Within a sense of *moral judgment*, they compared historical events with contemporary challenges: “Sometimes I catch myself comparing events from the present with what we saw in Auschwitz. I find these comparisons difficult, which are often made in the media (…), and yet it helps me to accept principles and form an opinion.” (48.6). Confronting the Holocaust led to questioning personally important values including religious belief for a student: “My naive religious faith in a paternalistic loving God was also shaken. (…) However, since my faith also represented a strong resource for me (…), Auschwitz left a dark hole on a spiritual level and led me into an identity crisis.” (46.6). Importance of prioritizing moral values was expressed as a strong sense of *moral motivation*: “Making people afraid has such a great power. That's why I think it's very important to stand by your values and not always go along with what the group does or says.” (40.6). The meaning of moral character was expressed by referring to moral courage, “Above all, I have noticed that my inner courage to stand up for these things has increased.” (7.5), ego strength: “This trip is another stone in my inner wall against fascism, against the attack on human dignity.” (49.6), and responsibility: “I also noticed a certain sense of responsibility, even for situations in which I was perhaps not directly personally involved but in which injustice occurred.” (43.6). Reinforced attitude was also expressed: “My stance against racism and persecution was already there before, but it was reaffirmed by the visit to Auschwitz.” (24.6). These considerations were described as leading to intended actions: “(…) taking a step out of that speechlessness and to actively do my part to ensure that something like this never happens again.” (2.6) and realized moral actions such as *speaking up *or *engaging in discourse*, “Discrimination in language and deeds used to freeze me; now I feel more liberated to enter into dialogue. To seek confrontation. More constructive.” (1.5), *empathy and caring*: “I pay more attention to people and to what they say. (…) at the same time more loving when it comes to trying to understand the person opposite me.” (1.5), as well as *further reading*: “Specifically, I deal with the Nazi era much more than before. On the one hand, I do this consciously by reading a book that deals with this time (…).” (48.6). Those reporting moral outcomes also reflected on their actions as a starting point for intensified future action: “This does not always work, but I believe that through my own reflection and awareness I can become better and better at it.” (50.6) (see also table 2 (Tab. 2)).

#### 3.2.2. Theme 2: Curriculum and study trip impact on me as a student and future health professional

Students also described how the curriculum and study trip affected morality components within actual study conditions, patient encounters and aspects of PIF. Increased *moral sensitivity* included upholding values in patient care was described, ie. “I believe that the trip has made me more alert and sharpened my senses for degrading behavior towards patients in a medical context, but also in everyday situations” (43.6) as well as *moral judgment* in regard to treatment of underrepresented patient groups: “I observe changes regarding a critical questioning of my own attitude especially in relation to the treatment of migrants and also homeless people in the medical context.” (45.6). Students described enhanced moral motivation to prioritize *moral values*, ie. “Whenever I meet a new patient, I try to get to know them without prejudice and with an open heart and open perception” (50.6) as well as aspects of *moral character* to campaign for these values: “(…) the excursion showed me once again the importance of these values and the need to stand up for them.” (44.6). Students also reported *intended actions* as future healthcare practitioners: “I have also set myself the goal of traveling to crisis areas in the course of my professional career and providing medical care for those in need” (41.6) as well as* moral outcomes* such as *speaking up*, “Even in everyday hospital life, unfortunately, there are always situations in which “slight” discrimination or racism appear. In such situations, I always mustered up my courage and protested against them” (41.6) and enhanced *caring*: “There was a moment in the clinic when a doctor was very unkind and rude to a patient, this affected me greatly. I felt a strong sense of injustice and thought I should do something about it. When the doctor left the room and I stayed behind with the patient, I apologized to the patient and tried to give her some sympathetic words along the way. I didn’t manage to point out his misconduct to the doctor (…), but at least I managed not to remain completely inactive in the situation.” (7.6) (see also table 3 (Tab. 3)).

#### 3.2.3. Theme 3: Curriculum and study trip impact on me as a member of a health profession (medicine/psychology)

Students reflected on the role of the medical profession in history and contemporary healthcare. They reported increased *moral sensitivity* for that role in the past including the issue of dehumanization, “I am still preoccupied with the fact that medicine played a central role in the dehumanization and ultimately the subsequent atrocities and crimes” (7.6) and today: “Dehumanization, however, is something that we as physicians often see in the medical context as well. (…) In the case of the homeless person who regularly shows up in the emergency room, we forget even more quickly that this is a fellow human being and not an object. (…) But at the core of all of them is the same dehumanization that made Auschwitz-Birkenau possible.” (45.6). One student reflected on challenges within moral decision-making (*moral judgment*): “It was brought home to me once again how doctors today also have to make major ethical and moral decisions in therapy on a daily basis. We are often not prepared for these decisions.” (19.6). Medical practice system conditions were questioned: “It seems to me that especially in a system that does not create any framework conditions for interpersonal dignity and in which there can be hardly any talk of healing, it is only possible to treat patients with human dignity (…) if an inner attitude is repeatedly developed” (51.6). The *motivation* to act morally and to fight for moral principles not only by single persons, but by all physicians was also described: “I find it even more important to always keep the principles of medicine, the prohibition to harm, the principle of autonomy and the principle of human dignity, in mind and to fight with all means to implement these principles. Not only by me but by all physicians.” (33.6). Responsibility as part of collective *moral character* of the medical profession in society was highlighted: “The key element that I took away from the study trip and the discussions connected with it is the question of collective responsibility of the individual, both as a doctor and as a human being.” (1.6). As a result, one student described how he/she become actively *engaged in social action*, namely aid to medical refugees: “After the trip, I started to work in the medical refugee aid. We make sure that everyone gets the right to medical care whether he is registered in Germany or not (is paperless).” (50.6) (see also table 4 (Tab. 4)).

#### 3.2.4. Theme 4: Curriculum and study trip impact on me as global citizen and part of humanity

*Moral sensitivity* in the context of societal and existential questions was described and principles of free democratic societies were reflected: “I think the vigilance has the most effect on me. I am very aware of what freedom means and that EVERYONE has the right to experience (…).” (50.6). A student also discussed prisoners’ resistance and became aware that the planned dehumanization of people failed: “(…) the study trip showed me that the prisoners were not only victims who surrendered to their situation. There was also resistance. This was not necessarily visible in the situation at the time, but it shows us retrospectively that the Nazis’ plan to dehumanize Jewish prisoners failed.” (23.6). Statements expressing *moral judgment* included reflections on Jewish life in Germany today: “If people of Jewish faith leave Germany nowadays again because they have to fear to become victims of violence and attacks, then this is a cruel proof that not enough is done for their protection. And even if this protection would be guaranteed by the authorities, do people want to live like this? Under police protection?” (49.6). *Moral motivation* was expressed in the impulse to become politically active: “I see the study trip as the starting point of my personal politicization in medicine. (…) it became clear to me that I absolutely have to politicize myself as a private person and as a (future) doctor and learn to take a stand.” (5.6) as well as to protect democracy: “I think it became even clearer to me that a democracy is something fragile, something special, and therefore one must protect it well.” (24.6). Statements expressing *moral character* included the role of resistance and ego strength: “The Nazi era shows like no other - and especially the setting of Auschwitz - what each individual can contribute to the system, in every respect: be it by unquestioned consequences or by willfully turning a blind eye. It also shows what resistance is still possible despite adverse circumstances. In any case, it shows: nobody is irrelevant.” (1.6). A statement of* intended action* included a future task of strengthening moral character: “For me, it is a future task to awaken human values, to strengthen the human ‘Me’ and to do a lot of awareness work - especially for the roots.” (20.6) (see also table 5 (Tab. 5)).

This RW quote summed up the essence of the work: “I want us humans to learn from history (…).” (40.6).

### 3.3. Comparison between RWs 6 weeks and 1.5 year after the study trip

Students’ narratives of RW5 were generally briefer and content differences between RW5 and RW6 were noted. Not all students attended the post-trip course meeting for RW5 or submitted their RW. RW5 content was focused more on moral sensitivity regarding personal and global citizenship aspects. Narratives contained more “living the questions” expressed in question marks with reasoning and meaning-making appearing to be at a more preliminary stage, e.g. “And what does it matter if one questions such beliefs. Acts upon them. And if one asks (and questions) again and again, who is this person who says this, to say this to myself. Why are they saying it? And that I am free in my choice to accept it or not.” (37.5). RW6 narratives contained more descriptions of students’ actual moral behavior in specific clinical situations. These descriptions were generally lengthier and more elaborated, not generally found in RW5 and suggested motivation and follow-through at 1.5-2 years post study trip to speak up, engage in discourse, and bring a more caring approach towards patients.

## 4. Discussion

### 4.1. Overview

Moral development including moral sensitivity on the path to moral competence is a key element of PIF yet cultivating and sustaining this can be challenging. Our prior research on health professions students’ RWs during a four-day study trip to Auschwitz as part of a MNH curriculum revealed intention to use learning for moral courage and responsibility [11]. Within our current study of longitudinal outcomes of this curriculum, the RW content led to our exploring the longitudinal impact of learning and reflecting on this history on moral development within personal and PIF. Within this group of students, we found that exploring history-informed PIF [12] included exploring what we term “history-informed moral development” in HPE. 

Our analysis of the students’ RWs revealed impact of the curriculum including the study trip on actual moral behavior, with examples provided of going beyond intended action to actualizing it. At a more sophisticated level as per theoretical frameworks of Kegan [4] and Kohlberg [65], students describe finding their own agency or “moral compass” with an overall moral vigilance activated (with reflection) when encountering “disorienting” dilemmas or challenging situations [57]. This aligns with Kegan’s self-authored professional [4] who constructs a discerning principled identity [66]. Such moral agency for human dignity can be stress-buffering and potentially counteract moral distress to promote well-being which can then positively impact PIF [67]. Students’ writings in relation to self, profession, healthcare system, and existentially, to humanity, included aspects of moral competence, ie. four components of moral sensitivity, judgment, motivation, and character as well as actual behavior.

RWs indicated a sense of awareness and moral commitment fostered by the curriculum but also its facilitating “speak-up culture” [68] and taking moral action when needed within situations generating moral distress that can and do occur in the clinic and other educational experiences. They describe how standing up to injustice can be challenging, even hard within power hierarchies and competing priorities in HPE and healthcare. This seems to be a challenge not only for the medical and psychology professions. Formative guidance to foster moral courage and practice in undergraduate nursing education has been recommended in regard to keeping quiet or speaking up in ethically challenging nursing care situations [69]. 

Students writing about the need for “collective responsibility” (1.6) and how “democracy is something fragile, something special, and therefore one must protect it well” (24.6) for example, embodies the concept of responsibility and identity existing in symbiosis with each other [70]. Developing moral sensitivity, judgment, motivation, and character as part of one’s personal and professional identity construction helps sustain a moral compass in general as well as specifically within moral dilemmas and challenges. This is relevant for fostering medical professionalism (for which PIF serves as foundational) for the “moral contract between the doctor and the individual patient in society” ([71], p. 1619). Our conceptual framework combines two theoretical frameworks to frame and contain RW content [19], [20], [63] and captures general moral vigilance and awareness generated by experiencing disorienting or challenging situations (see figure 1 (Fig. 1)).

Moral behavior that our group of students described as a result of the curriculum and trip experience included speaking up, engaging in discourse, empathy and caring, engaging in social action, intentionality and perseverance, and further reading. This aligns with identity formation being entwined with moral virtues and values [72], supporting trainees toward the highest stage of identity formation, ie. a person who chooses, internalizes, and lives by good moral values [4], for healthcare and global citizenship. 

Many RWs captured moral integrity as active and intentional. A direct, significant relationship between moral development, “ethical sensitivity” and greater empathy was reported in medical students ([73], p.73) which may mitigate “ethical erosion”, be protective against burnout [74] and contribute to high quality clinical care. Our curriculum (with formative elements of reflective writing and narratives, emotional aspects of learning, and guiding reflection on moral implications) and study trip [11] may thus serve as a “preventive” or proactive approach for this and for avoiding distortion of moral agency within inherent risks for abuse in medicine, thus enhancing moral resilience in PIF [4]. Such risks include obedience within hierarchy in the profession [75] as well as inevitable temptations or economic, professional, and/or political pressures. Students grapple with the implications for their personal and PIF of “doctors and other health professionals being among the most avid of Nazi ideologues with their abandonment of moral principles resulting in an unprecedented breach of ethics” ([75], p.105). 

Working with biographies before and during the site visit, preparation for and debriefing the site visit as well as interactive reflective writing sessions and reflection about the medical profession during the site visit helped to increase the awareness of the relevance of the content and appeared to be an important curriculum element for self-reflection, as reported by the students. This is in line with the reported value of reflective discussions for working professionals at site visits [50], reflective process reportedly facilitating undergraduate students’ perceived knowledge acquisition and awareness of the Holocaust after visiting the Auschwitz concentration camp with the reflection enabling empathic connection between these historical events and the students [76], and medical students’ statements on initiating reflection and learning processes relevant for their future professional practice [53].

Analysis of RW5 at six weeks and at 1.5-2 years post-study trip (RW6) revealed awareness and sensitivity in RW5 with a developmental process moving from intended action to realized action (seen in RW6). We can perceive six weeks as a “segue” with learners still processing and relevance emerging more clearly with experience. In essence, it can take some time within immersion in one’s HPE including experiencing challenging situations to recognize meaningful impact of such a curriculum and study trip for professional growth. We noted sustained impact of the curriculum with “deepening” through reflection on this history, for example, with greater sensitivity to unjust treatment in healthcare, reflecting on Jewish life including concern about dangers associated with contemporary antisemitism, and ongoing “living the questions” with connections to daily life. In this vein, Horton noted how the value of teaching the Holocaust in medicine includes providing “a powerful societal bulwark against antisemitism” ([75], p.105). 

Within the students’ RWs in general, we identified components of transformative learning [58] including a disorienting experience, an emotional response, critical reflection; perspective change; and a commitment to future action [77] and furthermore, actual action. Our analyses were informed by Mezirow’s transformative learning work including its definition “as the process by which we transform problematic frames of reference (mindsets, habits of mind, meaning perspectives) – sets of assumption and expectation – to make them more inclusive, discriminating, open, reflective and emotionally able to change.” ([78], p.92). As such, the transformative character of students’ reflections was demonstrated by phrases such as “My self-image has changed (…)” (14.6), “I am much more aware (…)” (2.6), “I have definitely become more sensitive (…)” (42.6), “I observe changes regarding a critical questioning of my own attitude (…)” (45.6), or “I see the study trip as the starting point of my personal politicization in medicine.” (5.6). 

In accordance with the definitions of Frenk and colleagues of the Lancet Commission on Medical Education for the 21st Century [79], transformative learning is accompanied by informative and formative aspects of learning in this study. The study outcomes reflect “informative” within learning the history, “formative” work within the data emerging in the RWs, and “transformative” within the students referencing the change in themselves which we can term as “enlightened change agents” using Frenk and colleagues’ terminology [79]. 

We also noted confirmatory learning [58] outcomes within some RWs with students describing strengthening and reaffirming of their moral convictions resulting from the study trip which may contribute to bolstering moral resilience and/or courage as needed. 

Study findings help realize aims of the Lancet Commission on MNH, ie. to “support the development of morally conscious and self-critical, yet courageous and resilient health professionals – independent thinkers who are capable of upholding professional values in the face of pressure and who will, when needed, act as agents of change” ([12], p.1867) and be “history-informed morally courageous health professionals who will speak up when necessary” ([80], p.1817). The students in their RWs connect the dots between learning this history and their learning and further developing moral awareness, sensitivity, and action, components of history-informed personal and PIF as discussed in the report of the Lancet Commission [12]. Our results also align with a recently published study on learning outcomes for a medicine during the Holocaust curriculum and study trip to Auschwitz for U.S. medical students [45].

Our findings advance the field of supporting PIF in HPE as our data supports the use of curriculum (with critical reflection) on history of MNH with a study trip component to cultivate moral development within personal and PIF in HPE with longitudinal impact.

### 4.2. Limitations

Limitations of our study include some suboptimal elements of RW5 session with scheduling issues and some students unable to attend, and no behavioral indicator for real life moral behavior. Data was solely from students’ voluntary RWs after the study trip and obtained from one institution in one country (Germany) with self-selected students for the module, thus raising a question of transferability. 

An inherent limitation is the risk of social desirability within empirical social research based on self-reports. High expectations of a visit to a memorial site, ie. “expectation production” may engender a risk of potentially overshadowing the actual learning effect [49]. Recommended measures [81] were taken within our study to counteract this phenomenon, ie.

1. Expectations and motivations for visiting the memorial were discussed with the students prior to the visit and on the first evening of the visit,
2. Anonymity was ensured in the RWs, as was sufficient response time for the open-ended prompts, and
3. There was no dependency of the students on the researchers either before or after the course, ie., in exams or other forms of assessment.

Not all students submitted RWs when invited to do so for this study. Our response rate of 52% however is noted as above average in academic studies [82]. Our number of analyzed RWs (n=34) is in range with studies analyzed in a review of students’ RW in the context of learning (range 8-880, Median 32) [83].

Students’ level of training, gender, and/or cultural background would be of interest in future studies as would examples of application of knowledge, skills, and attitudes gleaned from this curriculum within residency training and beyond. Our findings within the current longitudinal study revealed MNH curriculum with study trip serving as an enriching experience for transformative learning, moral development, and history-informed PIF. Further longitudinal studies are needed to obtain additional insights on history-informed transformative learning within personal and PIF of health professions students. 

## 5. Conclusions

Analysis of our data suggests that a MNH curriculum with study trip has the potential to catalyze a critically reflective learning/meaning-making process with longitudinal impact to support moral development within personal and PIF including critical consciousness, moral awareness, and professional value. Guiding a reflective process of grappling with the implications of the difficult history of physician atrocities during Nazism and the Holocaust as well as deriving inspiration from moral courage and resistance narratives can help foster moral development with a sense of responsibility for moral leadership. Such history-informed PIF can be foundational for emergence of empathic healthcare professionals bringing moral sensitivity and moral character to realize humanistic healthcare.

Medical ethicist Dr. Edward Pellegrino’s observation rings true as we consider the results of our study: “Medicine is a moral enterprise” ([84], p.65). Within this, we conclude with Mary Lagerway’s citing philosopher Emmanuel Levinas, that “Encountering the face of the Other calls forth a response of moral sensitivity to one’s responsibility to the Other” ([85], p.614).

## Acknowledgements

The authors wish to thank Diethard Tauschel, MD, director of the curriculum program, Prof. Dr. med. Peter Selg, Ita Wegman Institute for Basic Research into Anthroposophy, Switzerland, Dr. phil. Krzysztof Antonczyk, Auschwitz-Birkenau Memorial and Museum, Poland for facilitating the study trip and educational presentations, and to all the students who participated. 

## Notes

### Ethics approval

This study was approved by the Ethics Committee of the University Witten/Herdecke with participants informed that voluntary submission of their anonymous reflective writings would be used in this study.

### Authors’ contributions

The study design and research question were developed by all researchers. MSR collected the RWs from the students. CK and MSR analyzed the data. HSW acted as an external audit of the data analysis. All authors contributed to the planning and writing of the manuscript. HSW developed and facilitated the interactive reflective RW sessions during and after the excursion.

### Author’s ORCID

Claudia Kiessling: [0000-0003-4104-4854]

## Competing interests

The authors declare that they have no competing interests. However, HSW serves as a commissioner, the Lancet Commission on medicine, Nazism, and the Holocaust. The views presented herein do not necessarily represent the other members of the Lancet Commission on medicine, Nazism, and the Holocaust, but rather the views of the author only.

## Figures and Tables

**Table 1:**
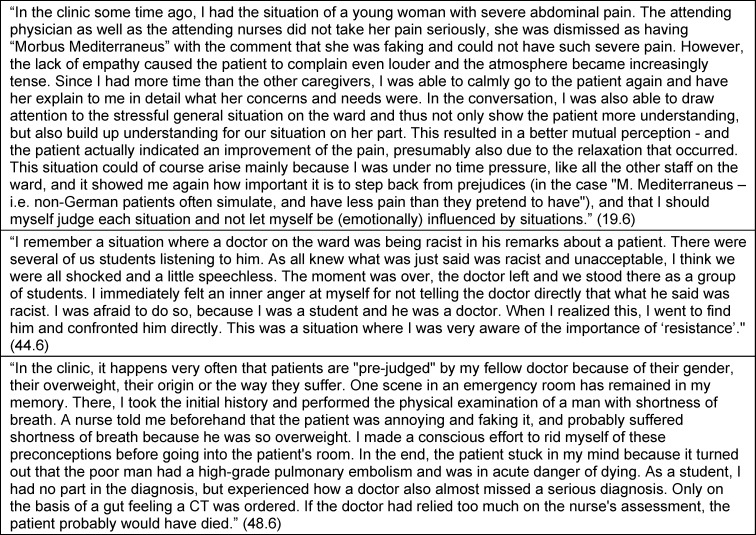
Examples of specific challenging situations in the clinical context described by students

**Table 2:**
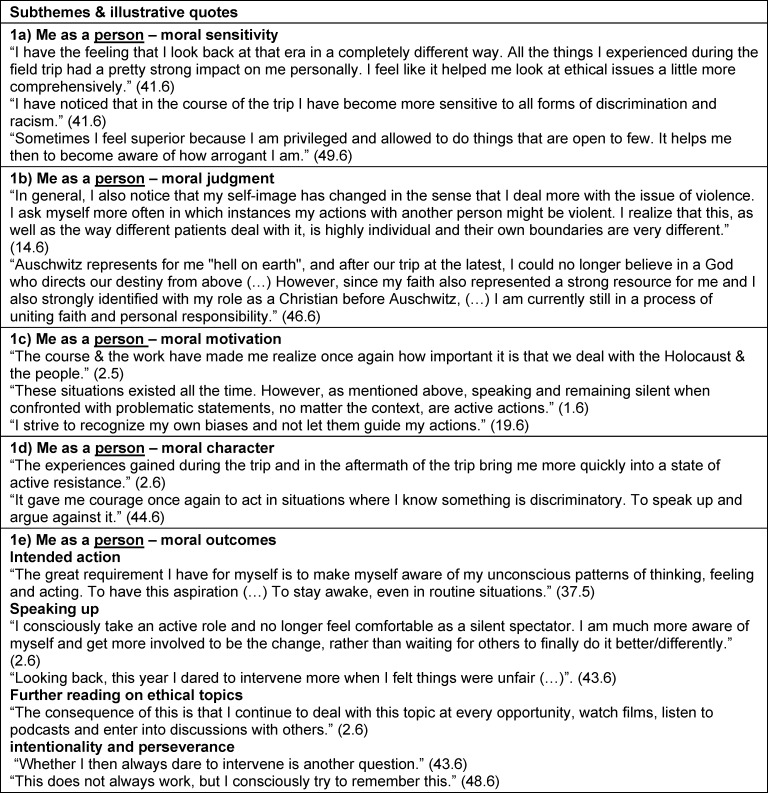
Theme 1 – Curriculum and study trip impact on me as a person

**Table 3:**
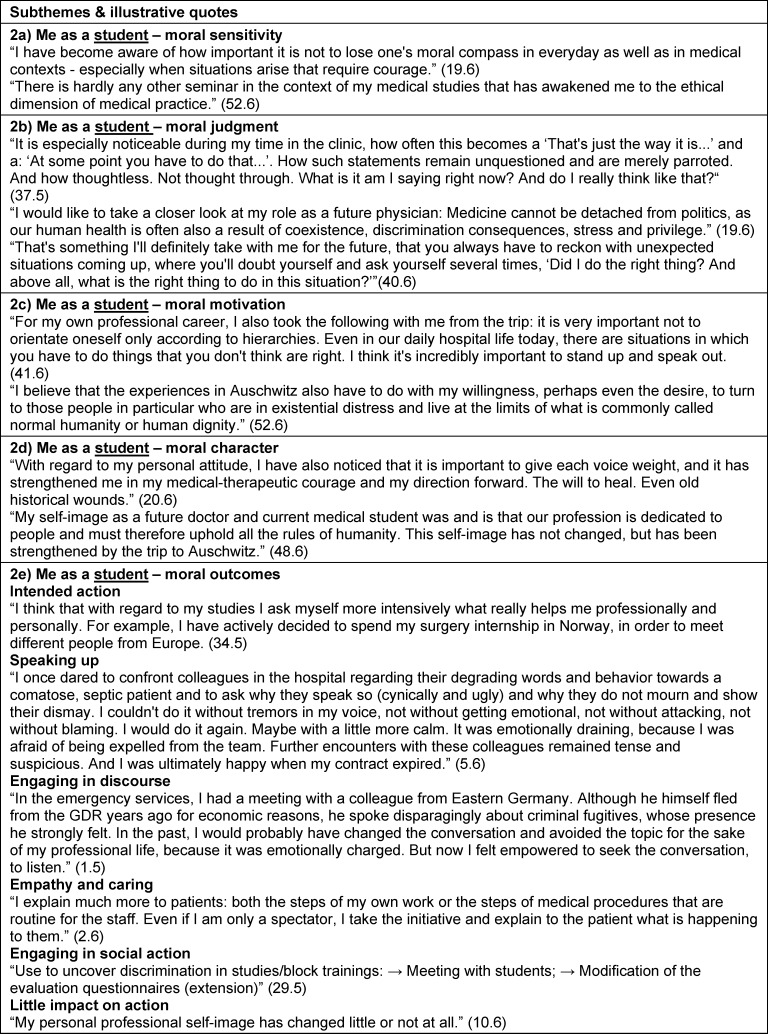
Theme 2 – Curriculum and study trip impact on me as *a student and future health professional including Professional Identity Formation *

**Table 4:**
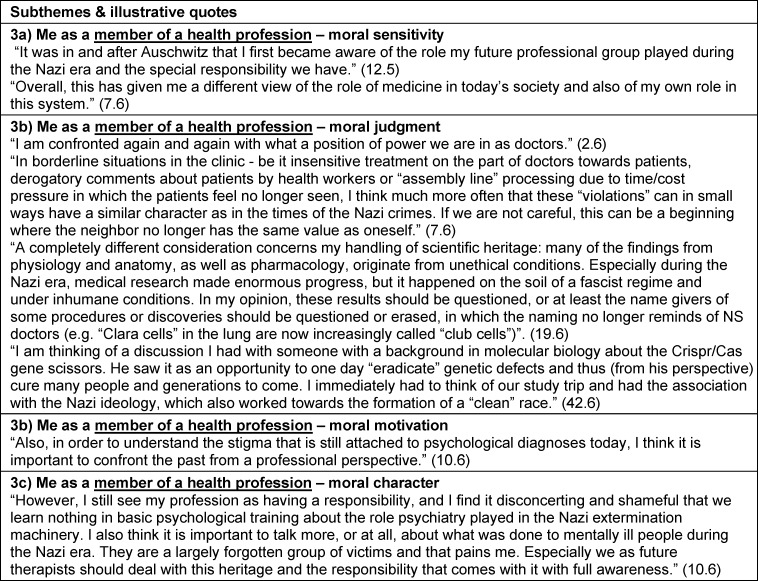
Theme 3 – Curriculum and study trip impact on me as a member of a health profession (medicine/psychology)

**Table 5:**
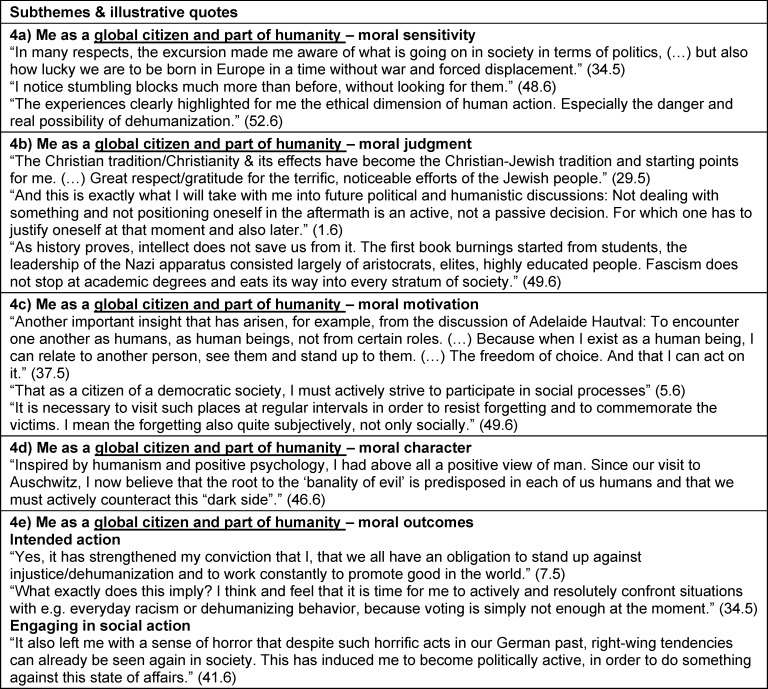
Theme 4 – Curriculum and study trip impact on me as a global citizen and part of humanity
